# Prevalence and associated factors of intertrigo in aged nursing home residents: a multi-center cross-sectional prevalence study

**DOI:** 10.1186/s12877-019-1100-8

**Published:** 2019-04-15

**Authors:** Sabrina Gabriel, Elisabeth Hahnel, Ulrike Blume-Peytavi, Jan Kottner

**Affiliations:** 1 Department of Dermatology and Allergy, Clinical Research Center for Hair and Skin Science, Charité-Universitätsmedizin Berlin, Berlin, Germany; 2 Department of Public Health and Primary Care, University Centre for Nursing and Midwifery, Ghent University, Ghent, Belgium

**Keywords:** Intertrigo, Prevalence, Nursing homes, Elderly, Risk

## Abstract

**Background:**

In geriatric and long-term care settings, intertrigo seems to be common, but generalizable epidemiological estimates are lacking. Aim of this study was to measure the prevalence of intertrigo in aged nursing home residents and to identify possible relationships with demographic and health characteristics.

**Methods:**

A cross-sectional prevalence study was conducted between September 2014 and May 2015 in a random sample of ten institutional long-term care facilities in Berlin, Germany. In total 223, aged long-term care residents were included. Mean age was 83.6 (SD 8.0) years and mean Barthel score was 45.1 (SD 23.8). Board certified dermatologists and study assistants performed skin assessments and measurements according to standard operating procedures. Mean differences and odds ratios between residents with and without intertrigo were calculated.

**Results:**

The prevalence of intertrigo was 16.1% (95% CI 11.6 to 21.2%). The submammary fold was most often affected (9.9%), followed by the inguinal region (9.4%), axilla (0.5%) and abdominal region (0.5%). Increased age was statistically significantly associated with the presence of intertrigo (OR 1.05; 95% CI 1.00 to 1.10). Care dependency in bathing activities was associated with intertrigo. Obesity, sex and skin functional parameters were not associated with intertrigo.

**Conclusions:**

Every sixth nursing home resident was affected by intertrigo indicating the high load of this skin condition in this population. Older age seems to be associated with intertrigo. Care dependency in bathing activities was likely to be associated with intertrigo. Structured skin care regimens are needed to prevent and treat intertrigo in this population.

**Trial registration:**

This study is registered at https://clinicaltrials.gov/ct2/show/NCT02216526. Registration date: 8th November 2014.

## Background

Intertrigo or intertriginous dermatitis is an inflammation of skinfolds, caused by occlusive conditions and skin-on-skin friction. It is most often associated with secondary bacterial or fungal infections and a pathogenic flora (e.g., *Staphylococcus aureus* or *Candida albicans*). Intertrigo is initially characterized by mild erythema that initially manifests itself as red plaques [1]. Severe forms are accompanied by itching, burning sensation, pain and odor. The diagnosis is based on clinical inspection, potassium hydroxide preparation, or fungal culture [2]. Intertriginous dermatitis generally affects the inguinal, perineal, axillary and submammary folds and can affect patients throughout their lives [1, 3–5].

There are only few empirical studies describing the epidemiology of intertrigo. In a prospective study in two dermatology units in Senegal, the prevalence of intertrigo was reported to be 2.5% for adults with an average age of 41.5 years [6]. In geriatric hospital patients, nursing home residents and homecare clients reported prevalence estimates range from 4 to 20% [3, 5, 7–9]. Recently, based on skin inspections by nurses the Dutch National Prevalence Measurement of Quality of Care reported a prevalence of 6.7% in nursing home residents and 11% in homecare clients [7]. Using a similar measurement approach the prevalence of intertrigo in Austria varies from 3.6% in nursing home residents to 9.1% in geriatric hospital patients [8]. However, until today there was no systematic dermatological assessment of intertrigo in aged long-term care residents in Germany.

A number of individual and environmental factors have been associated with this condition. Especially obese people are assumed to be at high risk [10–14]. Other associated factors include incontinence, hyperhidrosis, poor hygiene, malnutrition and increased age [1, 3]. Furthermore, it is generally believed that this condition is more common in patients with diabetes mellitus [15, 16].

Ageing in general is associated with a number of cutaneous changes such as an increase of the skin surface pH and a decrease of the skin barrier function increasing the susceptibility to a wide range of age related skin conditions [17, 18]. For instance, the increase of the skin surface pH promotes candida skin infection. The skin pH in intertriginous regions of diabetic patients was reported to be significantly higher than in healthy subjects [16, 19]. Skin dryness is also a common problem in aged and geriatric patients [20] which is often associated with decreased stratum corneum hydration (SCH) [21, 22] and decreased transepidermal water loss (TEWL) [22, 23]. However, whether these intrinsic skin changes or other demographic and functional characteristics are associated with intertrigo in geriatric populations is unclear.

The main objectives of this study were to measure the prevalence of intertrigo in aged long-term care residents and to identify possible relationships with demographic and health characteristics.

## Methods

### Study design and setting

The data used in this study are from a descriptive, observational, cross-sectional prevalence study, conducted between September 2014 and May 2015 in a random sample of ten institutional long-term care facilities in Berlin, Germany. Detailed methods have been previously described [24–26].

### Ethics approval and consent to participate

The study was approved by the ethics committee of the Charité-Universitätsmedizin in Berlin (EA1/190/14). Informed consent was obtained from the residents themselves or their legal representatives prior any study procedure.

### Participants

At participating residential care facilities, all residents who were 65 years or older and who gave their informed consent, personally or via a legal representative, were considered eligible. Residents at the end of life were excluded.

### Variables

The following variables were measured: age, sex, duration of residency (months), body mass index (BMI, kg/m^2^) and concomitant diseases according to the ICD 10. The Barthel Index was used to measure care dependency, which generates a total score from 0 (full dependence) to 100 (independence) [27]. Mobility was measured with the respective Barthel index item and residents were classified as ‘completely dependent’, ‘wheelchair independent’, ‘some help’ and ‘without help’. Based on the Barthel index item ‘bathing’, residents were classified into ‘bathing with some help’ and ‘bathing without help’ [27–29]. The overall dry skin score (ODS) was used to measure skin dryness. The degree of dryness was classified from ‘0’ (no skin dryness) to ‘4’ (advanced skin roughness, large scales, inflammation and cracks) [30]. Pressure ulcer (PU) risk was assessed with the Braden scale [31]. Three skin functional parameters were measured in triplicates at the right inner midvolar forearm. TEWL is a measure of the flux density of condensed water, which diffuses through the stratum corneum to the skin surface [23, 32]. It was measured with the Tewameter TM300 (Courage + Khazaka Electronic, Cologne, Germany). Stratum corneum hydration (SCH) was measured with Corneometer CM825 (Courage + Khazaka), skin surface pH was measured with the skin-pH-Meter PH905 (Courage + Khazaka) and the skin surface temperature was measured with Skin-ThermometerST500. It is the strongest predictor for TEWL. Therefore, TEWL values were adjusted to a standard skin surface temperature of 30 °C [33].

### Data sources/measurement

At the days of the data collection dermatologists conducted head-to-toe skin examinations. Trained study assistants, who examined and interviewed the participating residents and extracted data from the medical records, collected data about demographics, care dependency and medical history.

### Bias

All nursing homes of the state of Berlin were randomly selected to reduce selection bias. Board certified dermatologists and trained study assistants used validated tools to perform all assessments and measurements according to standard operating procedures.

### Study size

A formal sample size calculation was not performed. It was expected that the point estimate of proportions vary widely, therefore a prevalence of 0.5 of skin diseases was assumed. To measure this proportion with a 95% confidence interval (CI) width of ±0.06, 280 subjects were regarded as sufficient [24, 34].

### Quantitative variables

A BMI higher than or equal to 30 kg/m^2^ was categorized as obesity according to the WHO [35]. Underweight was defined as BMI of less than 18.5 kg/m^2^. For this analysis only the ODS of the trunk was considered and the score was dichotomized into ODS 0 to 1 (absent skin dryness or faint scaling, faint roughness and dull appearance) and ODS 2 to 4 (small or larger scales; roughness; redness, cracks and eczematous changes possibly).

### Statistical methods

All analyses were conducted using the Statistical Analysis System SPSS (Version 22.0. Armonk, NY: IBM Corp.). Descriptive statistics such as mean and spread parameters and proportions were computed to describe the characteristics of the nursing home residents. Mean differences including 95% CIs were calculated for continuous variables comparing residents with and without intertrigo. Odds ratios (ORs) including 95% CIs were calculated to compare residents with and without intertrigo for binary variables. Because the duration of residency was non-normally distributed, the Mann Whitney U test was used. *P*-values lower than 5% (two-sided) and 95% CIs different from 1 were considered statistically significant. ORs below 0.5 and above 2.0 were considered likely to be associated. P-values and CIs were considered descriptively. Because of the exploratory approach of the study the main focus is based on strength and directions of associations instead of statistical significance.

## Results

### Participants

Ten out of 55 contacted nursing homes agreed to participate. In total, 811 residents were assessed for eligibility, out of which, 252 subjects provided written informed consent. Prior examination 29 residents declined participation. Finally, 223 subjects were included.

### Descriptive data

Sample characteristics are shown in Table 1. The percentage of women was 67.7%, mean age was 83.6 (SD 8.0) years and the BMI was 25.3 kg/m^2^ (SD 5.1). The median duration of residency was 27 months. Regarding mobility, 21.6% were completely dependent, 23.4% wheelchair independent, 32.0% needed some help and 23.0% lived without help. Circulatory systemic diseases were diagnosed in 82.5% and endocrine, nutritional and metabolic diseases in 54.7% of all participants. The prevalence of intertrigo was 16.1% (95% CI 11.6 to 21.2%). The submammary fold was most often affected (9.9%), followed by the inguinal region (9.4%), axilla (0.5%) and abdominal region (0.5%). The temperature adjusted mean TEWL was 10.4 (SD 7.2) g/m^2^/h, mean SCH was 41.2 (SD 9.5) and mean pH was 5.1 (SD 0.6).

**Table 1:** Characteristics of participants (*n* = 223)

Age, years	
Mean (SD)	83.6 (8.0)
Median (IQR)	84.0 (78.0–89.0)
Female, *n* (%)	151 (67.7)
Body mass index (kg/m^2^)^a^	
Mean (SD)	25.3 (5.1)
Median (IQR)	24.6 (21.9–28.3)
Obesity BMI ≥ 30 kg/m^2^, *n* (%)	35 (15.7)
BMI 18.5–29.9 kg/m^2^, *n* (%)	167 (74.9)
Underweight BMI < 18.5 kg/m^2^, *n* (%)	14 (6.3)
Barthel Index total score^b^	
Mean (SD)	45.1 (23.8)
Median (IQR)	45.0 (25.0–65.0)
Barthel Index - Bathing^b^	
Some help, *n* (%)	204 (91.5)
Without help, *n* (%)	18 (8.1)
Barthel Index - Mobility^b^	
Completely dependent, *n* (%)	48 (21.5)
Wheelchair independently, *n* (%)	52 (23.3)
Some help, *n* (%)	71 (31.8)
Without help, *n* (%)	51 (22.9)
Braden score^b^	
Mean (SD)	17.3 (3.7)
Median (IQR)	18.0 (14.0–21.0)
Duration of residency (months)	
Mean (SD)	42.6 (49.1)
Median (IQR)	27.0 (14.0–52.0)
Intertrigo, *n* (%)	36 (16.1; 95% CI 11.6 to 21.2)
Intertrigo submammar	22 (9.9)
Intertrigo inguinal	21 (9.4)
Intertrigo abdominal	1 (0.5)
Intertrigo axilla	1 (0.5)
Overall Dry skin score (ODS) – trunk^b^	
ODS trunk 0–1, n (%)	144 (64.6)
ODS trunk 2–4, n (%)	78 (35)
TEWL arm, temperature adjusted (g/h/m^2^)^b^	
Mean (SD)	10.4 (7.2)
Median (IQR)	8.6 (7.3–10.2)
Stratum corneum hydration arm^b^	
Mean (SD)	41.2 (9.5)
Median (IQR)	40.8 (35.1–47.1)
pH arm^c^	
Mean (SD)	5.1 (0.6)
Median (IQR)	5.1 (4.7–5.5)
Common concomitant diseases, ICD-10 system level 1, n (%)	
Diseases of the circulatory system (I.00–I.99)	184 (82.5)
Mental and behavioural disorders (F.00–F.99)	157 (70.4)
Endocrine, nutritional and metabolic diseases (E.00–E.99)	122 (54.7)
Diseases of the genitourinary system (N.00–N.99)	106 (47.5)
Diseases of the nervous system (G.00–G.99)	99 (44.4)
Diseases of the musculoskeletal system and connective tissue (M.00–M.99)	96 (43.0)

*ICD*-10 International Coding of Diseases classification, *IQR*, Interquartile range, *SD* Standard deviation, *TEWL* Transepidermal water loss

^*a*^*n* = 216, ^b^*n* = 222, ^c^*n* = 221

### Main results

Comparisons between subjects with and without intertrigo are shown in Table 2. Females were more frequently affected by intertrigo compared to males (OR 1.290 (95% CI 0.585 to 2.842)). An increased risk for the presence of intertrigo was found with increasing age (OR 1.052 (95% CI 1.004 to 1.102)). The highest mean difference between residents with and without intertrigo was also observed for age (3.2 (95% CI 0.3 to 6.0) years). Mean differences between BMI, care dependency, pressure ulcer risk, skin barrier characteristics TEWL, SCH and pH were minor. The proportion of skin dryness at the trunk was higher in residents with intertrigo (41.7%) compared to non-affected residents (33.7%). Needing help with bathing (OR 3.400 (95% CI 0.438 to 26.414)) and having a cardiovascular disease (OR 2.623 (95% CI 0.762 to 9.031)) were likely to be associated with intertrigo. Obesity, skin functional parameters, Barthel index and Braden score did not appear to be associated with the presence of intertrigo.

**Table 2:** Comparison of subjects with and without intertrigo

	No intertrigo *n* = 187	Intertrigo *n* = 36	Mean differences	OR
Age, years				
Mean (SD)	83.1 (8.1)	86.3 (7.4)	3.2 (95% CI 0.3 to 6.0)	1.052 (95% CI 1.004 to 1.102)
Female n (%)	125 (66.8)	26 (72.2)	n.a.	1.290 (95% CI 0.585 to 2.842)
Body mass index (kg/m^2^)^a,b^				
Mean (SD)	25.2 (5.1)	25.9 (5.2)	0.8 (95% CI −1.2 to 2.7)	1.029 (95% CI 0.961 to 1.101)
Obesity BMI ≥ 30 kg/m^2^, *n* (%)	29 (15.5)	6 (16.7)	n.a.	1.084 (95% CI 0.413 to 2.845)
BMI 18.5–29.9 kg/m^2^, *n* (%)	141 (75.4)	26 (72.2)	n.a.	0.820 (95% CI 0.355 to 1.890)
Underweight BMI < 18.5 kg/m^2^, *n* (%)	11 (5.9)	3 (8.3)	n.a.	1.449 (95% CI 0.383 to 5.485)
Barthel Index total score^a^				
Mean (SD)	44.9 (24.2)	46.1 (22.0)	1.2 (95% CI −7.1 to 9.5)	1.002 (95% CI 0.987 to 1.017)
Barthel Index - Bathing^a^				
Some help, *n* (%)	170 (90.9)	34 (94.4)	n.a.	3.400 (95% CI 0.438 to 26.414)
Without help, *n* (%)	17 (9.1)	1 (2.8)	n.a.	0.294 (95% CI 0.038 to 2.285)
Barthel Index - Mobility^a^				
Completely dependent, *n* (%)	42 (22.5)	6 (16.7)	n.a.	0.714 (95% CI 0.278 to 1.835)
Wheelchair independently, *n* (%)	43 (23.0)	9 (25.0)	n.a.	1.159 (95% CI 0.505 to 2.661)
Some help, *n* (%)	59 (31.6)	12 (33.3)	n.a.	1.132 (95% CI 0.528 to 2.428)
Without help, *n* (%)	43 (23.0)	8 (22.2)	n.a.	0.992 (95% CI 0.420 to 2.343)
Braden score^a^				
Mean (SD)	17.2 (3.8)	18.1 (3.2)	1.0 (95% CI − 0.2 to 2.2)	1.077 (95% CI 0.973 to 1.191)
Duration of residency (months)				
Median (IQR)	25.0 (13.0–52.0)	32.0 (15.3–54.0)	*p* = 0.34	
Overall Dry skin score (ODS) – trunk^b^				
ODS trunk 0–1, *n* (%)	123 (65.8)	21 (58.3)	n.a.	0.717 (95% CI 0.346 to 1.486)
ODS trunk 2–4, *n* (%)	63 (33.7)	15 (41.7)	n.a.	1.395 (95% CI 0.673 to 2.891)
TEWL arm (g/h/m^2^) temperature adjusted^a^				
Mean (SD)	10.7 (7.5)	9.0 (4.5)	−1.7 (95% CI − 3.6 to 0.2)	0.949 (95% CI 0.871 to 1.033)
Stratum corneum hydration arm^a^				
Mean (SD)	41.0 (9.9)	42.0 (7.0)	0.9 (95% CI −1.8 to 3.7)	1.010 (95% CI 0.973 to 1.049)
pH arm^a,c^				
Mean (SD)	5.1 (0.6)	5.2 (0.7)	0.1 (95% CI −0.2 to 0.3)	1.247 (95% CI 0.687 to 2.265)
Common concomitant diseases, ICD-10 system level 1, *n* (%)				
Diseases of the circulatory system (I.00–I.99)	151 (80.7)	33 (91.7)	n.a.	2.623 (95% CI 0.762 to 9.031)
Mental and behavioural disorders (F.00–F.99)	136 (72.7)	21 (58.3)	n.a.	0.525 (95% CI 0.251 to 1.097)
Endocrine, nutritional and metabolic diseases (E.00–E.99)	98 (52.4)	24 (66.7)	n.a.	1.816 (95% CI 0.858 to 3.845)
Diseases of the genitourinary system (N.00–N.99)	89 (47.6)	17 (47.2)	n.a.	0.985 (95% CI 0.482 to 2.013)
Diseases of the nervous system (G.00–G.99)	83 (44.4)	16 (44.4)	n.a.	1.002 (95% CI 0.489 to 2.055)
Diseases of the musculoskeletal system and connective tissue (M.00–M.99)	78 (41.7)	18 (50.0)	n.a.	1.397 (95% CI 0.684 to 2.857)

*ICD*-10 International Coding of Diseases classification, *n.a*. not applicable, *SD* Standard deviation, *TEWL* Transepidermal water loss

^a^*n* = 35 (intertrigo group), ^b^*n* = 181 (no intertrigo group), ^c^*n* = 186 (no intertrigo group)

## Discussion

### Key results

Based on a large randomly selected sample of aged residents in institutional long-term care facilities the prevalence of intertrigo was 16.1%. Our results suggest that the submammary and the inguinal regions are most often affected. Increasing age is statistically significantly associated with intertrigo. Care dependency in bathing activities was likely to be associated with intertrigo. Obesity was not associated with intertrigo.

### Limitations

A nonresponse and selection bias cannot be ruled out. Despite high recruitment efforts, only ten out of 55 contacted nursing homes agreed to participate. The anticipated sample size of 280 could not be achieved. Study results may be affected by selection bias, because participating and non-participating residential care facilities were different in terms of size and ownership and residents at the end of life were excluded. Diseases were not further specified, it is difficult to interpret the impact of diseases, for example between diabetes mellitus and intertrigo. Skin functional parameters were measured on extremities only, and not at skin areas predisposed to develop intertrigo (e.g. trunk). Because of the exploratory approach data interpretation was based on the strength and direction of associations and not on statistical significance. Therefore, this study is hypotheses generating. Due to the cross-sectional design, conclusions about causality cannot be made.

### Interpretation

This is the first study to investigate the prevalence of intertrigo among older residents in long-term care settings in Germany. Every sixth resident is affected by intertrigo indicating the high load of this condition in this vulnerable population. In previous studies in Austria and the Netherlands, the percentage of patients with intertrigo was lower [7, 8, 36]. A major advantage of the present study was the systematic dermatological assessment by board certified dermatologists who conducted head-to-toe skin examinations. Within the studies in Austria and the Netherlands nurses performed data collection and differences regarding the diagnostic accuracy are likely. The inclusion of aged nursing home residents (65+ years) is another difference. Previous studies indicated that obesity is associated with intertrigo [1, 3, 10, 11, 13, 14, 37]. The results of the current study do not support this finding. It is likely that in aged and care dependent populations other factors might be more important. In addition, the proportion of obese residents in our sample was much lower compared to the general population. For instance, the German Health Interview and Examination Survey for Adults reported a mean BMI of 29 kg/m^2^ for a 60- to 79-year-old population [38].

Increasing age was statistically significantly associated with intertrigo. Residents with intertrigo were approximately 3 years older than non-affected residents, which is clinically relevant. Chronological ageing is the strongest predictor for the “natural” course of skin ageing, termed intrinsic ageing [39]. This intrinsic ageing process increases the risk for skin conditions and cutaneous diseases [17]. Intertrigo might be one of these risks.

Mobility or dependencies in physical functions related to daily activities seem not to be related with intertrigo. However, although not statistically significant, results indicate an association with care dependency in bathing activities. The more the residents were care dependent in bathing activities, the higher might be the likelihood of the occurrence of intertrigo. Being more independent was protective. One possible explanation is that skin care is not optimal in preventing this skin problem [40]. Less attention or inadequate skin care interventions might increase the risk for intertrigo. These results are in accordance with previous study results, which showed associations between dry skin and skin care dependency [20, 41, 42]. It seems that skin care independency might be protective against skin problems in a nursing care environment. On the other hand, skin care dependency might also be an indicator for increased cutaneous vulnerability. Irrespectively from that, the finding emphasizes the potential of preventive skin care in healthcare settings [43].

In our sample the proportion of skin dryness at trunk was higher in residents with intertrigo (41.7%) compared to non-affected residents (33.7%). Even if these results are statistically not significant, they suggest that severe skin dryness, which is accompanied with an imbalance in the composition of stratum corneum components and a damaged skin barrier function [44], might be a risk factor for other skin conditions, such as intertrigo. Advanced age is a key risk factor for the development of skin dryness [20].

The results of TEWL, SCH or pH are similar to previous research [25]. Our study results indicate that the skin barrier characteristics TEWL, SCH and pH seem to have limited diagnostic value for intertrigo risk in aged 65 years and older.

### Generalizability

Because sample characteristics are similar to comparable studies conducted in this population [40, 41], we assume external validity of the study results.

Previous studies showed that mean TEWL on the midvolar forearms in aged 65 years and older were lower than in our study population [23, 32]. The reasons for these findings are unknown. It is known that TEWL is influenced by several factors. Therefore, it is to be assumed that TEWL is more a relative than an absolute characteristic [21, 32].

## Conclusion

Our data indicate a high load of intertrigo among older residents in long-term care settings in Germany. Older age seems to be associated with intertrigo. The current study do not support that obesity is associated with intertrigo. We observed that care dependency in bathing activities might be likely associated with intertrigo; a finding that emphasizes the importance of structured skin care regimens to prevent and treat intertrigo in this vulnerable population.

## Abbreviations

BMI: Body mass index
CI: Confidence interval
ICD-10: International Coding of Diseases classification
ODS: Overall dry skin score
OR: Odds ratio
PU: Pressure ulcer
SCH: Stratum corneum hydration
TEWL: Transepidermal water loss
